# Low levels of pyruvate induced by a positive feedback loop protects cholangiocarcinoma cells from apoptosis

**DOI:** 10.1186/s12964-019-0332-8

**Published:** 2019-03-12

**Authors:** Mingming Zhang, Yida Pan, Dehua Tang, Robert Gregory Dorfman, Lei Xu, Qian Zhou, Lixing Zhou, Yuming Wang, Yang Li, Yuyao Yin, Bo Kong, Helmut Friess, Shimin Zhao, Jian-lin Wu, Lei Wang, Xiaoping Zou

**Affiliations:** 1 Department of Gastroenterology, Nanjing Drum Tower Hospital, the Affiliated Hospital of Nanjing University Medical School, Nanjing University, No.321 Zhongshan Road, 210008 Nanjing, People’s Republic of China; 2 Key laboratory of Reproduction Regulation of NPFPC (SIPPR, IRD), Fudan University, Shanghai, 200032 China; 3 Department of Digestive Diseases of Huashan Hospital, Shanghai, China; 4 School of Life Sciences, Fudan University, Shanghai, China; 5 Northwestern University Feinberg School of Medicine, Chicago, IL USA; 6 Department of Gastroenterology, Nanjing Medical University Affiliated Drum Tower Clinical Medical College, Nanjing Medical University, Nanjing, China; 7 Department of Surgery, Technical University of Munich (TUM), Munich, Germany; 8 State Key Laboratory of Quality Research in Chinese Medicine, Macau Institute for Applied Research in Medicine and Health, Faculty of Chinese Medicine, Macau University of Science and Technology, Avenida Wai Long, Taipa, Macao 442000 People’s Republic of China

**Keywords:** cMyc, Pyruvate, HDAC3, Apoptosis, Cholangiocarcinoma

## Abstract

**Background:**

Cancer cells avidly consume glucose and convert it to lactate, resulting in a low pyruvate level. This phenomenon is known as the Warburg effect, and is important for cell proliferation. Although cMyc has often been described as an oncoprotein that preferentially contributes to the Warburg effect and tumor proliferation, mechanisms of action remain unclear. Histone deacetylase 3 (HDAC3) regulates gene expression by removing acetyl groups from lysine residues, as well as has an oncogenic role in apoptosis and contributes to the proliferation of many cancer cells including cholangiocarcinoma (CCA). HDAC inhibitors display antitumor activity in many cancer cell lines. Cancer cells maintain low levels of pyruvate to prevent inhibition of HDAC but the mechanisms remain elusive. The purpose of our study was to explore the role of cMyc in regulating pyruvate metabolism, as well as to investigate whether the inhibitory effect of pyruvate on HDAC3 could hold promise in the treatment of cancer cells.

**Methods:**

We studied pyruvate levels in CCA cell lines using metabolite analysis, and analyzed the relationship of pyruvate levels and cell proliferation with cell viability analysis. We cultivated CCA cell lines with high or low levels of pyruvate, and then analyzed the protein levels of HDAC3 and apoptotic markers via Western Blotting. We then explored the reasons of low levels of pyruvate by using seahorse analysis and ^13^C_6_ metabolites tracing analysis, and then confirmed the results using patient tissue protein samples through Western Blotting. Bioinformatics analysis and transfection assay were used to confirm the upstream target of the low levels of pyruvate status in CCA. The regulation of cMyc by HDAC3 was studied through immunoprecipitation and Western Blotting.

**Results:**

We confirmed downregulated pyruvate levels in CCA, and defined that high pyruvate levels correlated with reduced cell proliferation levels. Downregulated pyruvate levels decreased the inhibition to HDAC3 and consequently protected CCA cells from apoptosis. Synergistically upregulated LDHA, PKM2 levels resulted in low levels of pyruvate, as well as poor patient survival. We also found that low levels of pyruvate contributed to proliferation of CCA cells and confirmed that the upstream target is cMyc. Conversely, high activity of HDAC3 stabilized cMyc protein by preferential deacetylating cMyc at K323 site, which further contributed to the low pyruvate levels. Finally, this creates a positive feedback loop that maintained the low levels of pyruvate and promoted CCA proliferation.

**Conclusions:**

Collectively, our findings identify a role for promoting the low pyruvate levels regulated by c-Myc, and its dynamic acetylation in cancer cell proliferation. These targets, as markers for predicting tumor proliferation in patients undergoing clinical treatments, could pave the way towards personalized therapies.

**Electronic supplementary material:**

The online version of this article (10.1186/s12964-019-0332-8) contains supplementary material, which is available to authorized users.

## Highlights

We found cMyc decreases pyruvate levels by promoting LDHA and PKM2 levels, this can consequently decrease the inhibition to HDAC3 and protect cancer cells from apoptosis. Conversely, high activity of HDAC3 stabilizes the cMyc protein by preferentially deacetylating cMyc at K323 site, which further contributes to low pyruvate levels. This creates a positive feedback loop that promotes the Warburg effect and cell proliferation of the tumor.

Together, this suggest the low pyruvate levels, regulated by c-Myc and its dynamic acetylation, can serve as a marker for predicting tumor proliferation in patients undergoing clinical treatments. These potential targets could pave the way towards personalized therapies.

## Background

Cancer cells mainly rely on aerobic glycolysis to generate enough energy and intermediates for the malignant behaviors. This so-called Warburg Effect can convert the glycolysis-induced pyruvate into lactate, thus making low pyruvate status in cancer cells [1]. The low pyruvate levels in cells are due to both reduced production and excessive consumption. The reduced pyruvate production comes from the expression of pyruvate kinase, the enzyme responsible for the generation of pyruvate in glycolysis. The splice variant PKM2 (musclespecific pyruvate kinase 2) is expressed specifically in cancer cells in the dimeric form with low catalytic activity, and is predictive of a poor prognosis in CCA patients [2]. The dominant pyruvate consumption comes from the conversion of pyruvate into lactate, and this reaction is mediated by lactate dehydrogenase (LDH). There are different isoforms of tetrameric LDH: LDHA and LDHB, and LDHA is effective in the conversion of pyruvate into lactate. Since tumor cells robustly convert pyruvate into lactate, one would expect a high expression of LDHA in cancer cells as well as cholangiocarcinoma (CCA) [3].

As an oncogene, *c-Myc* has attracted extensive interest as its potential role for contributing to tumorigenesis. *MYC*, and *c-Myc* in particular, is one such oncogene. *MYC* was discovered in studies of fulminant chicken tumors caused by oncogenic retroviruses. Subsequently, genomic sequencing efforts identified *c-Myc* as one of the most highly amplified oncogenes in many different human cancers [4, 5]. There are various mechanism of MYC-induced tumorigenesis, including increased Warburg effect, and many studies have found that MYC increased metabolic proteins, such as LDH and PKM2 [6, 7]. Therefore, many studies focus on the therapeutic value of targeting Myc. So far, no small molecules can directly target c-Myc in vivo. Both suppressing c-Myc transcription by bromodomain inhibitors targeting BRD4 and destabilizing c-Myc protein level by SIRT2 inhibition significantly reduced cancer cell proliferation [5, 8]. As the stability of c-Myc contributed to tumorigenesis, additional studies have found that the stability of c-Myc protein is related to the low acetylation at K323 [9, 10]. The treatment of HDAC inhibitors (HDACi), but not SIRT inhibitors, induced c-Myc K323 acetylation as well as tumorigenesis inhibition, suggesting that at least one of HDACs is the deacetylase of c-Myc [11, 12]. Although cMyc have often been described as preferentially an oncoprotein that contributes to the Warburg effect and tumor proliferation, mechanisms of action still remain unclear.

Genetic or epigenetic alterations, which disrupt proliferation and cell death pathways, are the fundamental event for initiation and progression of cancer [13]. Imbalanced epigenetic networks have been identified in all types of cancers and involve multiple metabolic changes. Unlike genetic mutations, epigenetic modifications are potentially reversible, thereby allowing drugs acting on specific enzymes involved in the epigenetic regulation of gene expression and raising the possibility of epigenetic therapies [12, 14]. Among the epigenetic alterations, acetylation has emerged as a key post-translational modification and acetylases have been identified as key metabolic enzymes in cellular regulation [15]. Deacetylases are designated to four classes (I-IV), depending on their amino acid sequence structure [13]. Class I HDACs (1, 2, 3 and 8) play an important role in tumorigenesis and may be candidate targets for many cancer treatments [16, 17]. Recently, we confirmed high levels of HDAC3 expressed in CCA tissues, and found that this was associated with poor survival in CCA patients. Mechanistically, HDAC3 induced proliferation and protected CCA cells from apoptosis [18]. HDACs inhibitors have been noted for their ability to induce cell cycle arrest and apoptosis of a broad spectrum of cancer cells [13, 16–19]. Some agents have shown signs of efficacy in clinical trials. SAHA and romidepsin are U.S. Food and Drug Administration (FDA) approved for the treatment of cutaneous T-cell lymphoma [13]. Novel Class I HDACs inhibitors were found to be beneficial for tumorigenesis inhibition as well as the acetylation-induced cMyc degradation [18, 20–22].

Collectively, the mechanisms that underlie cMyc’s ability to promote tumorigenesis via the Warburg effect are poorly understood. Thus, the question of whether cMyc acetylation is beneficial for its induced-tumorigenesis still remains unanswered. Here we set out to identify whether low pyruvate levels regulated by c-Myc, and its dynamic acetylation, promote cancer cell proliferation. These targets, as markers of predicting tumor proliferation in patients undergoing clinical treatments, could pave the way towards therapeutic intervention in the treatment of CCA.

## Materials and methods

### Ethics, consent and permissions

All experiments utilizing animal and human samples were approved by the Ethical Committee of Medical Research, Nanjing Drum Tower Hospital, Affiliated Hospital of Nanjing University Medical School.

### Cell culture and reagents

One human intrahepatic biliary epithelial cell line HIBEpiC and six human cholangiocarcinoma (CCA) cell lines HuCCT1, OZ, HuH28, Hccc9810, RBE, and QBC939 were used. HIBEpiC were obtained from the ScienCell (ScienCell, CA, Carlsbad, USA). HuCCT1, OZ, HuH28, and Hccc9810 cells were obtained from the Japanese Collection of Research Bioresources (JCRB) (Tokyo, Japan). RBE cells were obtained from the Institute of Biochemistry and Cell Biology, Shanghai Institutes for Biological Sciences, Chinese Academy of Sciences (Shanghai, China). QBC939 cells were kindly provided by Professor Shuguang Wang from The Third Military Medical University (Chongqing, China). Cells were maintained in Dulbecco’s modified Eagle’s medium (DMEM) (Invitrogen, Carlsbad, CA, USA) containing 10% fetal bovine serum (Invitrogen), penicillin (Invitrogen) (100 U/ml) and streptomycin (Invitrogen) (100 U/ml). For apoptosis experiments, a moderate concentration of 1 mM ethyl pyruvate is present in order to maintain a proper basal apoptotic rate. Full-length HDAC1–3, PKM2, LDHA (wildtype) and cMYC (wildtype and K323R) plasmids were kindly provided by the Zhao lab of Fudan University (Shanghai, China). Cells were transfected with Lipofectamine 3000 (Invitrogen) according to the manufacturer’s protocol. The HDAC3 siRNA was commercially purchased from RiboBio (Guangzhou, China), siRNA-HDAC3–1: CCATGACAATGACAAGGAA, siRNA-HDAC3–2: GCATTGATGACCAGAGTTA, siRNA-HDAC3–3: GAATATGTCAAGAGCTTCA. HDAC3 shRNA (h) lentiviral particles were commercially purchased from Santa Cruz Biotechnology. RGFP966 (MCE, Monmouth Junction, NJ, USA) was commercially purchased.

### Cell viability assay

Cell viability was determined using the CCK-8 colorimetric assay in 96-well plates (2 × 10^3^ cells/well) (Dojindo, Minato-ku, Tokyo, Japan) and cultured at 37 °C with 5% CO2. After treatment at the indicated times, 10 ul of CCK-8 solutions was added to each well. Then, cells were incubated for one and a half hours. The absorbance of the samples at 450 nm was recorded using a scanning multi-well spectrophotometer. Relative cell viability (%) = (absorbance 450 nm of treated group - absorbance 450 nm of blank)/(absorbance 450 nm of control group − absorbance450 nm of blank) × 100.

### Cellular pyruvate levels assay

Cellular pyruvate levels were detected by using Pyruvate Colorimetric/Fluorometric Assay Kit (K609–100, Biovision, Milpitas, California, USA) following the manufacturer’s instructions.

### Western blot

Cells were lysed with 0.5% NP40 lysis buffer and proteins were blotted following standard protocol; except for the detection of acetylation, which used 50 mM Tris (pH 7.5) with 10% (*v*/v) Tween 20 and 1% peptone (AMRESCO, Solon, OH, USA) as a blocking buffer. Primary and secondary antibodies were diluted in 50 mM Tris (pH 7.5) with 0.1% peptone. Signals were probed using the chemiluminescence ECL plus reagent (Thermo, Grand Island, NY, USA) and detected using a Typhoon FLA9500 scanner (GE, Fairfield, CT, USA). Primary antibodies were as follows: HDAC1 (Abcam, Cambridge, UK), HDAC2 (Abcam), HDAC3 (Abcam), cleaved caspase-3 (CST, Danvers, MA, USA), cleaved PARP (CST), PARP (CST), PKM2 (Abcam), LDHA (Abcam), cMYC (Abcam), β-actin (Sigma), FLAG (Abcam), and HA (provided by the Zhao lab of Fudan University).

### Deacetylation assay

Cells were lysed in NP-40 buffer containing 50 mM Tris-HCl (pH 7.5) (Sigma, St Louis, MO, USA), 150 mM NaCl (Sangon, Shanghai, China), 0.5% Nonidet P-40 (Sigma), 1 μg/ml aprotinin (Sigma), 1 μg/ml leupeptin (Sigma), 1 μg/ml pepstatin (Sigma), 1 mM Na3VO4 (Sigma) and 1 mM PMSF (Sigma). For immunoprecipitation, 500 μl of cell lysate was incubated with HA antibody (provided by the Zhao lab of Fudan University) for 3 h at 4 °C with rotation. Then, 30 μl of Protein A Agarose (Millipore, Billerica, MA, USA) was added for 12 h at 4 °C with rotation, and the beads were washed three times with lysis buffer before proteins were dissolved in loading buffer. Deacetylation assays were carried out in the presence of 5 μg enzyme and 0.3 μg peptide in 30 μl reaction buffer [30 mM HEPES (Sigma), 0.6 mM MgCl2 (Sangon), 1 mM DTT (Sigma), 1 mM NAD+ (Sigma), 10 mM PMSF (Sigma)]. The deacetylation reaction was incubated for 2 h at 37 °C before the mixture was desalted by passing it through a C18 ZipTip (Millipore). The desalted samples were analyzed using a MALDI-TOF/TOF mass spectrometer (Applied Biosystems, Grand Island, NY, USA). The acetylated peptide used in the assay was TRKDYPAAK (Ac) RVKLDSVR (Glssale, Shanghai, China).

### Mitochondrial oxidative phosphorylation analysis

Oxygen consumption rate (OCR) was detected in real-time with the XF96 Extracellular Flux Analyzer from Seahorse Bioscience, Inc. (North Billerica, MA, USA) following the manufacturer’s instructions.

### Metabolite analysis

^13^C metabolism labeling experiments have previously been documented [23]. Metabolite extracts were collected and 2ul of metabolite extract samples were injected for the GC-MS analysis using an Agilent 6980 GC coupled to an Agilent 5973 MS system. Relative metabolite abundances were determined by normalizing abundances of each metabolite to the internal standard and to cell number.

### DEGs of paired-CCA from TCGA data

The CCA RNA-Seq data were downloaded from the TCGA database using The GDC Data Portal (https://portal.gdc.cancer.gov). The mRNA expression data included a total of 18 samples consisting of 9 normal sample and 9 paired-CCA samples. The sequencing data were all publicly available and no ethical issues were involved. The edgeR package in Bioconductor was used to screen the DEGs in CCA and normal liver tissue samples. The edgeR package is based on the negative binomial (NB) distribution, which can correct the overdispersion problem in RNA-seq data by using a Poisson model and a Bayes procedure. Data with expression values of zero were removed. The genes were deemed to be DEGs if |FoldChange| > 2, respectively, both with *p*-value < 0.01 and false discovery rate (FDR) < 0.05.

### Functional annotation

The Database for Annotation Visualization and Integrated Discovery (DAVID) online tool (https://david.ncifcrf.gov/) was used to conduct the functional and pathway enrichment analyses in our study. We performed GO and KEGG pathway enrichment analyses to detect the potential biological functions and pathways of the High and low expression genes in CCA.

### Immunohistochemistry

Tumor specimens from human and mice were fixed in 4% formalin and embedded in paraffin. Two to five human CCA tumor specimens from one patient were used for the IHC study. Standard procedures were followed for IHC except for the detection of targets. The staining intensity for each tissue was calculated by multiplication and the range of this calculation [24].

### Ubiquitination assay

Thirty-six hours following transfection, cells were lysed in 1% SDS buffer (Tris (Sigma), 0.5 mM EDTA (Sigma) and 1 mM DTT (Sigma), pH = 7.5), as well as boiled for 10 min. For immunoprecipitation, the lysates were diluted 10-fold in Tris-HCl buffer. Analyses of ubiquitination were performed using anti-HA blotting.

### CCA xenograft model

Nude mice were purchased from the Department of Laboratory Animal Science, Nanjing Drum Tower Hospital. HuCCT1 cells (5 × 10^6^) in FBS-free medium were subcutaneously injected into the abdomen of mice. The Animal Welfare Committee of Nanjing Drum Tower Hospital approved all procedures involving animals.

### Statistics

Data was expressed as means ± standard error of the mean (SE). The data was analyzed through one-way ANOVAs followed by post hoc Duncan tests (SPSS 17.0). *P* < 0.05 was considered significant.

## Results

### High levels of intracellular pyruvate inhibit the proliferation of CCA cells

**Fig. 1:**
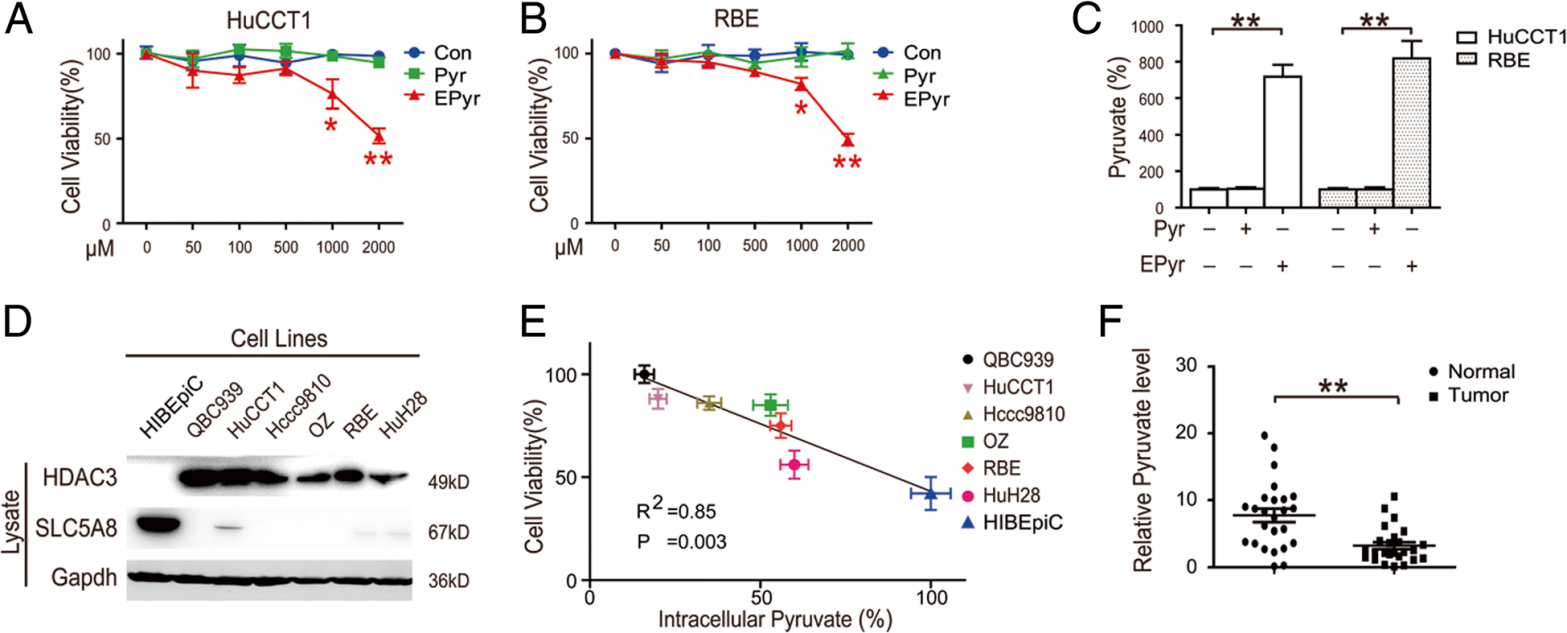
High levels of intracellular pyruvate in CCA cells inhibits its proliferation. **a** and **b** CCA cells were treated with pyruvate and ethyl pyruvate for 48 h, then quantified via CCK-8 assay. **c** CCA cells were treated as indicated for 48 h; the intracellular pyruvate was quantified and normalized to its protein level. **d** HDAC3 and SLC5A8 protein levels were detected by western blot. **e** The correlation between the cell viability and its intracellular pyruvate level in CCA cells. **e** Pyruvate levels in fresh tumor and adjacent normal tissues from 25 CCA patients were analyzed and normalized to its protein level. Data represent the Mean ± SEM, *n* ≥ 3. *p < 0.05, **p < 0.01, NS not significant

Previous studies have identified pyruvate as an HDAC inhibitor and indicate that tumor cells would silence Na + −coupled pyruvate transport SLC5A8, as well as convert pyruvate into lactate, as complementary mechanisms to avoid pyruvate-induced cell death [25]. Therefore, we examined the inhibitory effect of pyruvate on CCA. CCK8 assay results showed that marginal growth was inhibitory of pyruvate, even at levels as high as 2 mM in HuCCT1 cells and RBE cells (Fig. 1a & b). However, ethyl pyruvate, a stable lipophilic derivative of pyruvate which could penetrate the cell membranes directly, could significantly inhibit the proliferation of CCA (Fig. 1a & b). Consistent with the CCK8 assays, pyruvate levels in HCC cells were significantly increased only after ethyl pyruvate treatment (Fig. 1c). Our previous study confirmed that the highly-expressed HDAC3 was correlated with poor prognosis, and that HDAC3 inhibition is a promising anti-proliferation approach in CCA [18]. To further evaluate the relationship between intracellular pyruvate and CCA proliferation, we screened a normal bile duct epithelial cell line (HIBEpiC) and five human CCA cell lines (QBC939, HuCCT1, Hccc9810, OZ, RBE and HuH28). We found that CCA cells simultaneously have high levels of HDAC3 expression with low levels of SLC5A8 (Fig. 1d). Interestingly, we found that high levels of intracellular pyruvate negatively correlated with cell proliferation (Fig. 1e). Moreover, the low pyruvate levels in cells were further confirmed in fresh cholangiocarcinoma tissues (Fig. 1f). Collectively, these results reveal that high levels of intracellular pyruvate in CCA cells inhibits its proliferation.

### Pyruvate decreases the proliferation of CCA cells by inhibiting HDAC3

**Fig. 2:**
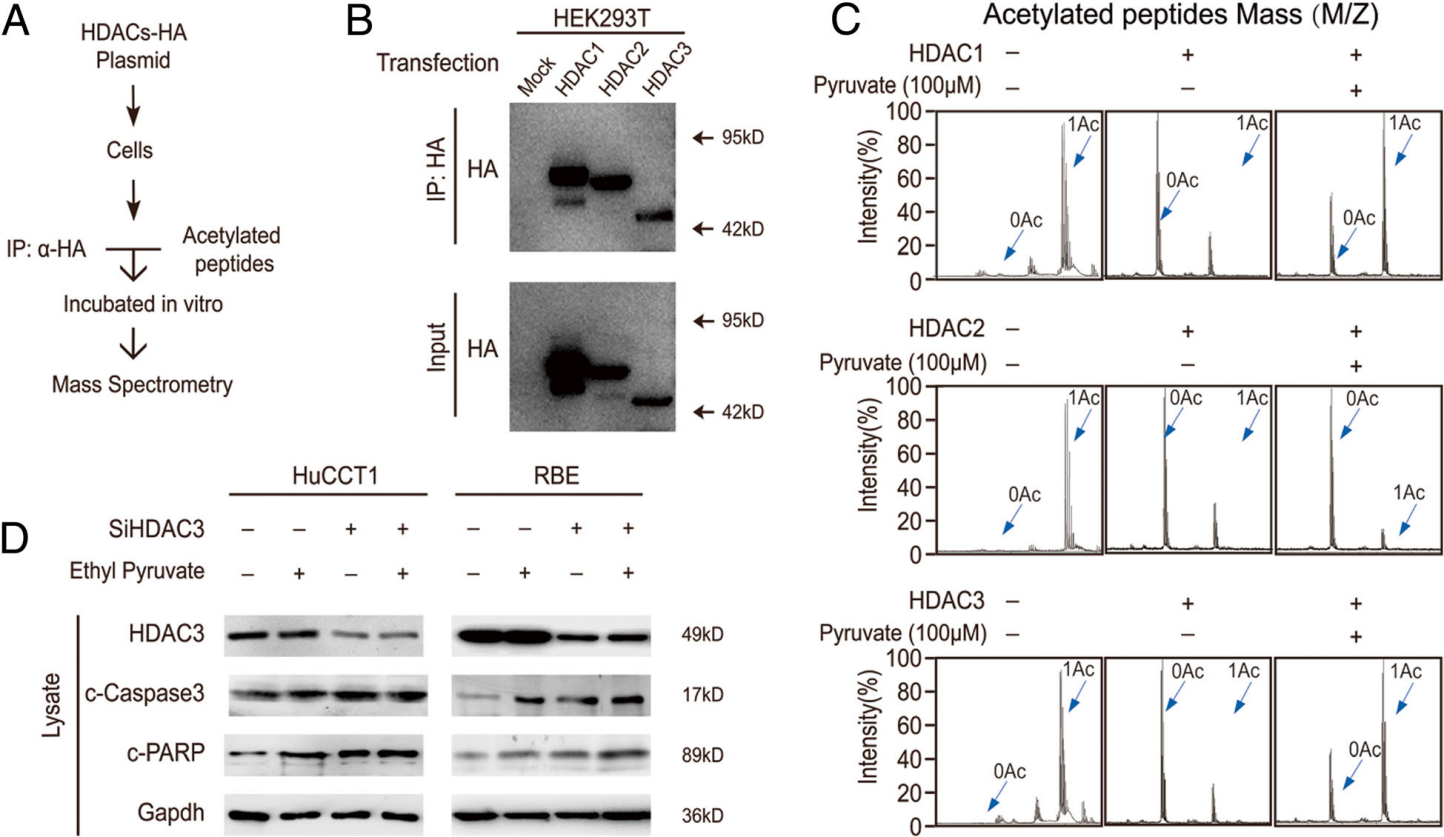
Pyruvate decreases the proliferation of CCA cells by inhibiting HDAC3. **a** Schematic diagram of the in vitro deacetylation assay with HDACs. **b** The immunoprecipitated protein corresponding to HDACs-HA was subjected to western blot. **c** The HDAC proteins were incubated with acetylated peptides with or without 100 μM pyruvate, and the rate of deacetylation was determined using Mass Spectrometry (MS). **d** HDAC3 knockdown cells and their counterparts were treated with 2 mM ethyl pyruvate for 48 h and subjected to western blot. Data represent the Mean ± SEM, *n* ≥ 3. *p < 0.05, **p < 0.01, NS not significant

Our previous data screened Class I HDACs and confirmed that only HDAC3 could rescue apoptosis in CCA cell lines [18]. Moreover, Class I HDACs shared a high level of homology with each other (HDAC 2 shares 52% identity with HDAC3) [26, 27]; thus, pyruvate possibly has a weak inhibitory effect on HDAC1 and 2 as well. To elucidate the direct target of pyruvate, we used an in vitro deacetylation system (Fig. 2a & b). Pyruvate treatment inhibited HDAC1 and 3 deacetylation activity, but only had a marginal inhibitory effect on HDAC2 (Fig. 2c). To determine if the anti-proliferative effect of ethyl pyruvate in CCA cells was due to HDAC3 inhibition, HDAC3-knockdown CCA cells were treated with ethyl pyruvate. HDAC3 protein levels were unchanged by ethyl pyruvate treatment, suggesting that ethyl pyruvate inhibits the activity of HDAC3 directly (Fig. 2d). Furthermore, ethyl pyruvate treatment significantly promoted cell apoptosis, whereas HDAC3-knockdown counterparts did not show significant apoptosis (Fig. 2d). These results demonstrate that HDAC3 is the main target of pyruvate in inducing CCA cell apoptosis.

### Changes in metabolic enzymes contribute to low levels of pyruvate

**Fig. 3:**
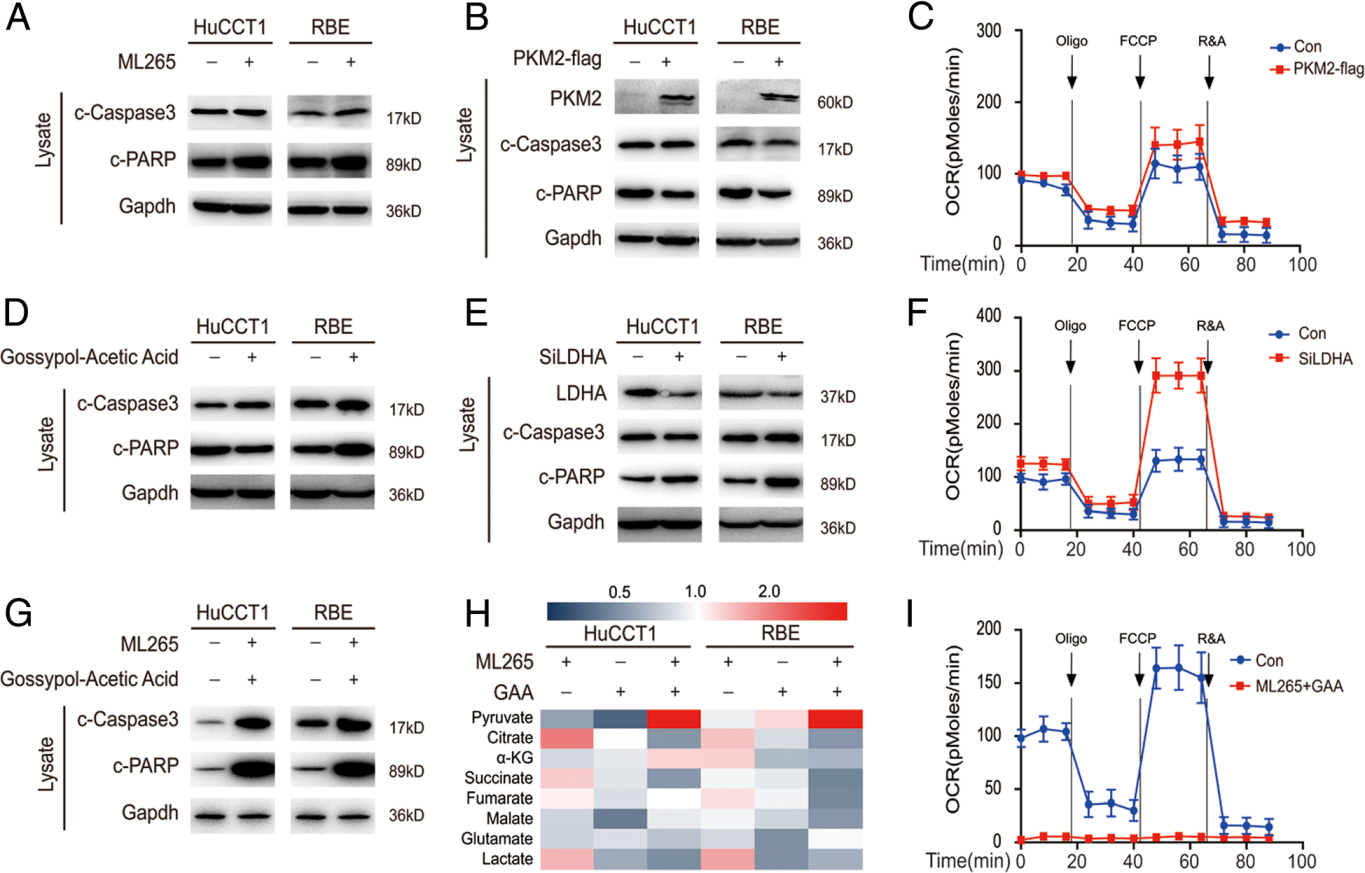
The changes in metabolic enzymes contribute to the low levels of pyruvate. **a** Cells were treated with 1 μM ML265 for 24 h and subjected to western blot. **b** PKM2 overexpressed cells and their counterparts were subjected to western blot. **c** The oxygen consumption rates (OCR) of PKM2-overexpressed cells and control cells were detected at different time points. OCR under oligomycin, carbonyl cyanide 4-(trifluoromethoxy) phenylhydrazone (FCCP), and antimycin A/rotenone treatments, respectively. **d** Cells were treated with 10 μM Gossypol-Acetic Acid (GAA) for 24 h and subjected to western blot. **e** LDHA knockdown cells and their counterparts were subjected to western blot. **f** The OCR of LDHA knockdown cells and control cells were detected at different time points. **g** Cells were treated with 1 μM ML265 and 10 μM GAA for 24 h, then subjected to western blot. **h** Cells were treated with 1 μM ML265 and 10 μM GAA for 24 h, the concentrations of the metabolites in GC cells were measured by Mass spectrometry (MS) and normalized to their protein level. **i** The OCR of cells were detected at different time points after they treated with 1 μM ML265 and 10 μM GAA for 24 h. Data represent the Mean ± SEM, *n* ≥ 3. *p < 0.05, **p < 0.01, NS not significant

One of the guaranteed anticancer strategies is to increase intracellular pyruvate levels [28], so we decided to further explore whether it is possible to promote pyruvate-induced apoptosis of CCA by promoting pyruvate generation. Pyruvate is generated in tumor cells principally by glycolysis. The splice variant PKM2 is expressed specifically in cancer cells in the dimeric form with low catalytic activity, and predicts a poor prognosis in CCA patients [2, 29]. ML265 (also called TEPP46) is a potent and selective activator of recombinant PKM2. Despite marginally increased oxygen consumption, both PKM2 activation and PKM2 overexpression did not significantly promote apoptosis of CCA cells (Fig. 3a, b & c). The dominant pyruvate consumption comes from the conversion of pyruvate into lactate. Gossypol-acetic acid (GAA) acts as an inhibitor for several dehydrogenase enzymes. This reaction is mediated by LDHA [3, 30, 31]. We found that LDHA knockdown increased oxygen consumption, and that both LDHA inhibition and LDHA knockdown did not significantly promote apoptosis of CCA cells (Fig. 3d, e & f). Due to the flexibility of pyruvate metabolism, targeting any of these metabolic enzymes could neither accumulate intracellular pyruvate levels nor promote apoptosis (Fig. 3a, d & h). Finally, we promoted the formation of pyruvate and reduced the degradation of pyruvate with two drugs, and we found that apoptosis of CCA cells was significantly increased (Fig. 3g). Accordingly, intracellular pyruvate levels were significantly increased, and oxygen consumption was extremely low, indicating that cells were on their way to apoptosis (Fig. 3g, i & Additional file 1: Figure S1A). These results suggest that low levels of pyruvate, resulting from changes in metabolic enzymes, protected CCA cells from apoptosis.

### cMYC leads to a change in metabolic enzymes in CCA cells

**Fig. 4:**
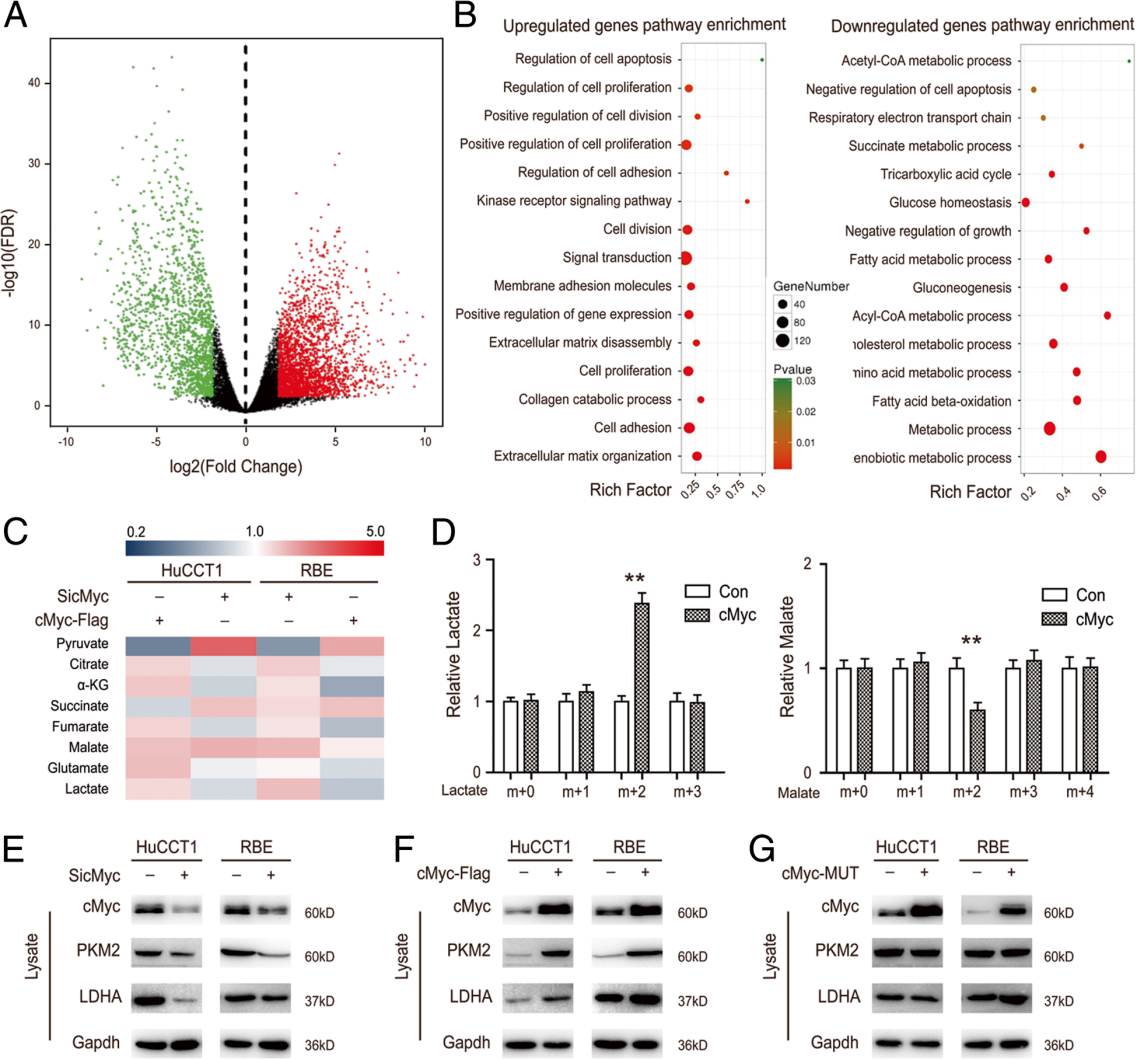
cMYC leads to a change in metabolic enzymes in CCA cells. **a** Heat map of differentially expressed genes (DEGs) in CCA and paired normal samples. The heat map was drawn using the gplots package in Bioconductor. DEGs with Fold Change (FC) > 2 were shown in red; DEGs with Fold Change (FC) < − 2 were in green (*P* < 0.01) and false discovery rate (FDR) < 0.05. **b** Gene Ontology (GO) annotation pathways of high and low expression genes in CCA. GO annotation pathways were generated using the ggplot2 package in R language. The size of the dots represents the number of genes. Dot color represents the *P*-Value. Red: high degree of enrichment, Green: low degree of enrichment. **c** After cells were transfected with the cMYC plasmid or SiRNA, the concentrations of the metabolites in the cells were measured by Mass spectrometry and normalized to their protein level. **d** ^13^C_6_ glucose labeling experiments were performed as described in the Methods. cMYC-overexpressed and control HuCCT1 cells were treated with ^13^C_6_ glucose for 1 h, then relative abundance of ^13^C_3_ lactate and ^13^C_4_ malate was determined by gas chromatography-mass spectrometer (GC-MS). The incorporation of ^13^C atoms are denoted as m + n, where n is the number of ^13^C atoms. **e** Cells were transfected with cMYC SiRNA and subjected to western blot. **f** Cells were transfected with the cMYC plasmid and subjected to western blot. **g** Cells were transfected with the mutant cMYC plasmid and subjected to western blot. Data represent the Mean ± SEM, *n* ≥ 3. *p < 0.05, **p < 0.01, NS not significant

Tumors can change their metabolic status through differential gene expression, thereby promoting their malignant biological behavior [32]. In order to find the upstream target of changes in pyruvate metabolism, we downloaded the CCA RNA-Seq data from the TCGA database using The GDC Data Portal (https://portal.gdc.cancer.gov), which consisted of 9 normal samples and 9 paired-CCA samples. We confirmed the differentially expressed genes and then performed KEGG pathway enrichment analysis (Fig. 4a). Moreover, we observed that the most relevant pathways were metabolic and apoptosis pathways in CCA (Fig. 4b). These results suggest that the upstream gene-keeper that regulates the expression of metabolic enzymes may be principal for pyruvate metabolism in CCA. Previous studies have shown that *c-Myc,* as an oncogene, has a role in contributing to tumorigenesis in many different human cancers [4, 5]. There are various mechanisms for cMYC-induced tumorigenesis, including an increased Warburg effect, and many studies have found that MYC increases metabolic proteins, such as LDHA and PKM2 [6, 7]. Therefore, we focused on the pyruvate metabolism with cMYC. Metabolite detection demonstrated that cMYC induced a similar metabolism pattern to drug (ML265 and GAA) treatment, and this significantly increased intracellular pyruvate levels (Figs. 3k & 4c). Pyruvate is generated in tumor cells principally by glycolysis and glutaminolysis. The levels of pyruvate in these cells are controlled not only by these two metabolic pathways, but also by the cells’ ability to convert pyruvate into lactate [28]. To further identify if the pyruvate-accumulating effects of cMYC comes from glycolysis, we utilized ^13^C_6_ glucose tracings and subsequent mass spectrometry. We found that cMYC overexpressed cells exhibited a marked increase in ^13^C_3_-labeled lactate as well as a significant decrease in ^13^C_2_-labeled malate. These results suggest cMYC-induced glycolysis contributed to the low pyruvate status (Fig. 4d). The expression of *cMYC* differs in different cancers [33, 34], contributing to an abnormal metabolic status. We found cMYC knockdown simultaneously decreased the levels of PKM2 and LDHA, resulting in high intracellular pyruvate levels, as well as that cMYC overexpression could reversed it (Fig. 4c, e & f). Moreover, the mutant type of cMYC had no effect on the levels of PKM2 and LDHA (Fig. 4g). Together, these results indicate that cMYC-induced pyruvate metabolic reprogramming is contributed to by changes of glycolytic enzymes in CCA cells.

### cMYC-induced metabolic enzyme changes contribute to a poor prognosis in CCA patients

**Fig. 5:**
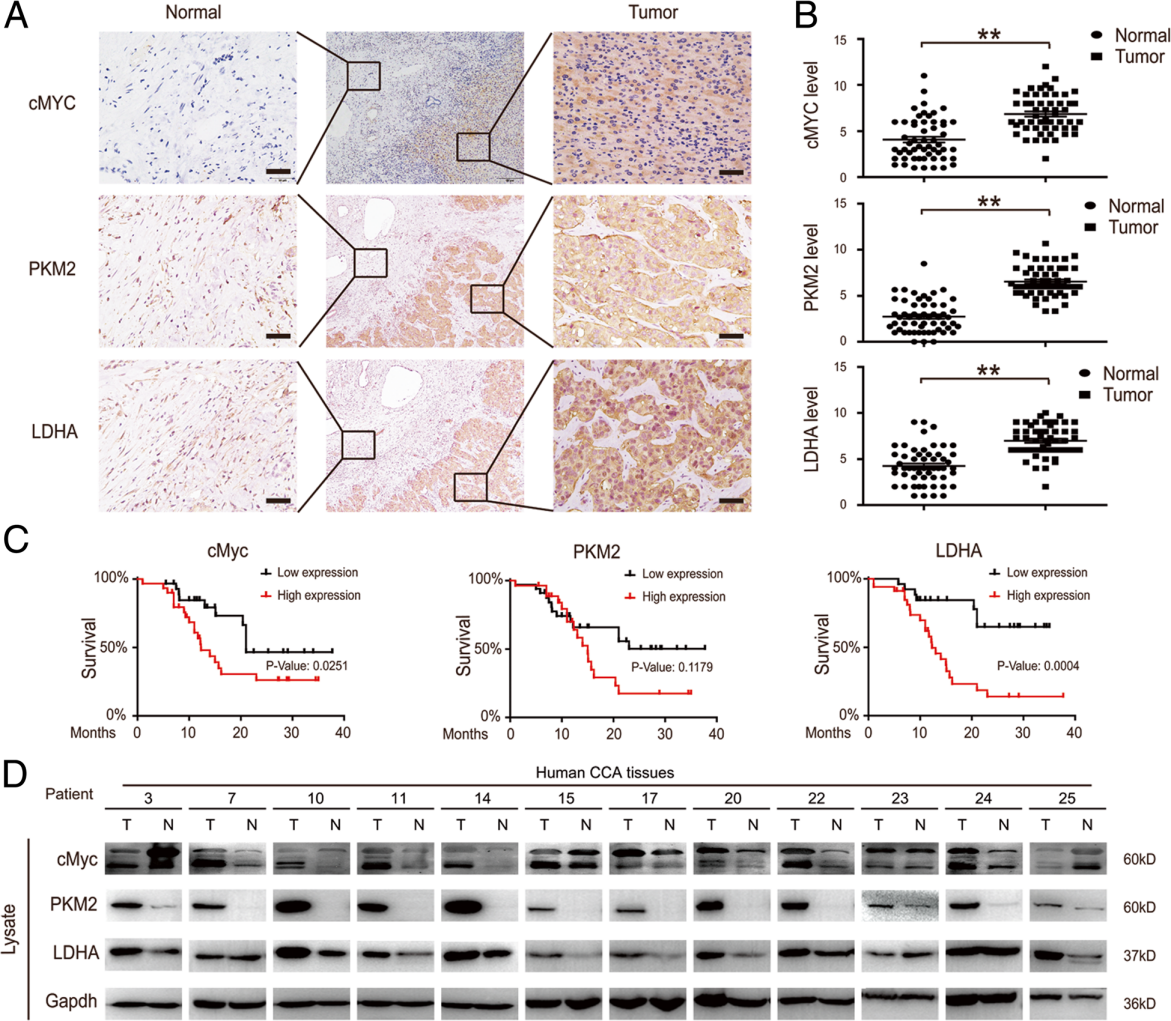
cMYC-induced metabolic enzyme changes contribute to a poor prognosis in CCA patients. **a** and **b** The indicated protein levels in tumor and adjacent normal tissues from 60 CCA patients were analyzed by IHC (left) and quantified (right). Scale bars, 100 μm. **c** 3-year survival was evaluated for CCA patients with the indicated protein levels. Data represent the mean ± SEM, *n* ≥ 3. *p < 0.05, **p < 0.01. **d** The indicated protein levels in fresh tumor and adjacent normal tissues from 25 CCA patients were analyzed by western blot. Data represent the Mean ± SEM, *n* ≥ 3. *p < 0.05, **p < 0.01, NS not significant

PKM2 and LDHA expression is upregulated in CCA samples, and their expression correlates with tumor recurrence and outcome [2, 31, 35]. We used CCA tissues that contain clinically annotated data from 60 CCA samples; the clinical characteristics of participants are summarized in Table 1. Consistent with previous studies, we found that the level of biological targets (cMYC, PKM2 and LDHA) were increased in CCA tissues as compared to adjacent tissues (Fig. 5a & b). Importantly, these increased biological targets were associated with a reduced patient survival (Fig. 5c). These results were further confirmed by an immunoblotting assay in fresh tissues (Fig. 5d). Of note, pyruvate levels in fresh tissues were negatively correlated with an increased cMYC level (Fig. 1f & Additional file 1: Figure S1B). Collectively, these results revealed that cMYC-induced metabolic enzyme changes contribute to a poor prognosis in CCA patients.

### HDAC3 deacetylates cMYC at K323 and protects cMYC from ubiquitinated degradation

**Fig. 6:**
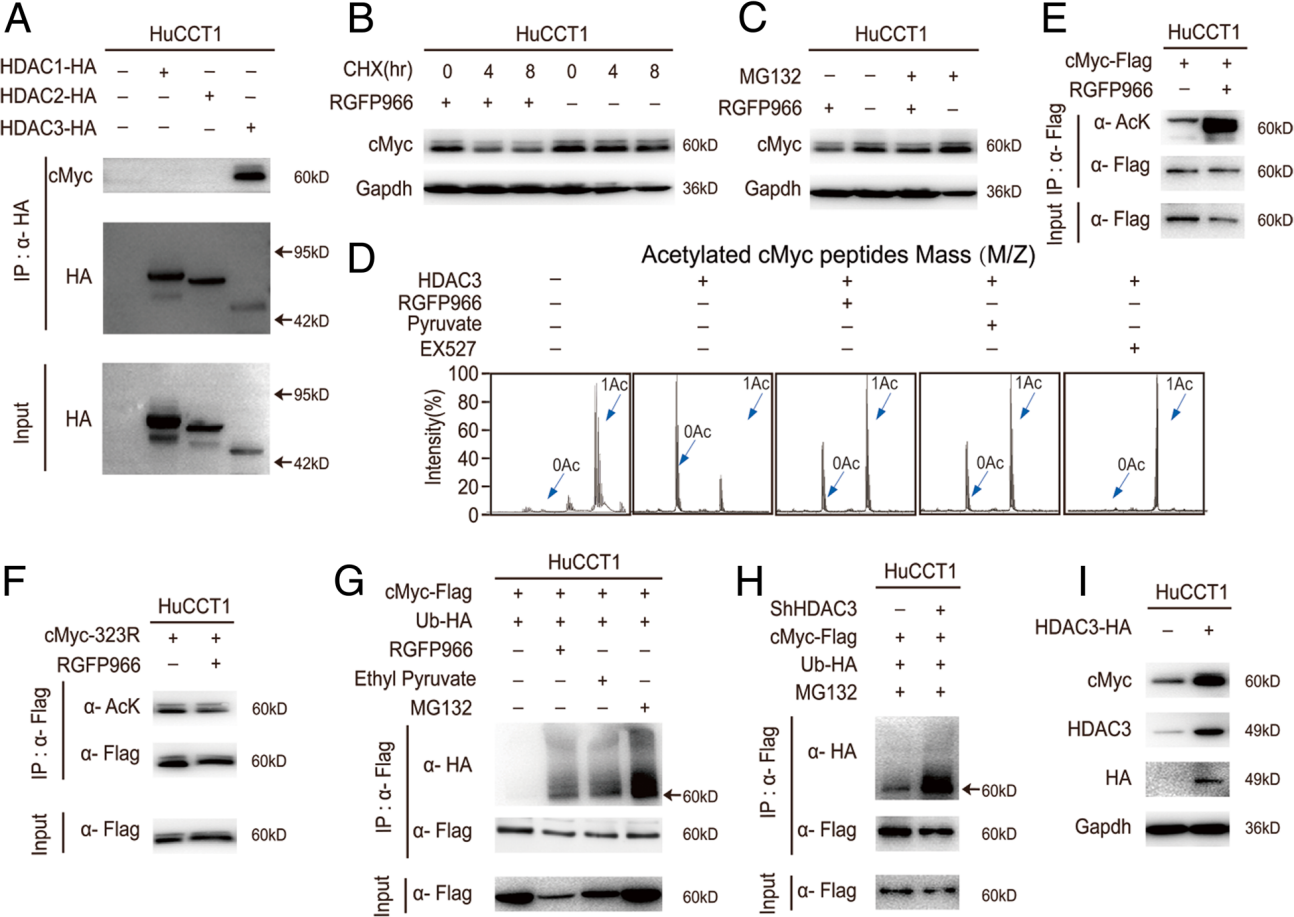
HDAC3 deacetylates cMYC at K323 and protects cMYC from ubiquitinated degradation. **a** HuCCT1 cells were transfected with HA-tagged HDAC1–3 plasmid for 36 h, then cells were lysated and immunoprecipitated with HA-beads. The input was visualized by Western blotting with the specific antibody. The immunoprecipitated sample was washed and visualized by Western blotting with the antibody for HA and cMYC. **b** HuCCT1 cells treated with 100μg/ml CHX (Cycloheximide), with or without 10 μM RGFP966 for 6 h. Cells were lysated and visualized by Western blotting. **c** HuCCT1 cells treated with 10 μM MG132 with or without 10 μM RGFP966 for 24 h. Cells were lysated and visualized by Western blotting. **d** HDAC3 deacetylated toward cMYC (K323) acetylated peptides were assayed in the presence of 10 μM RGFP966 and 100 μM pruvate. The deacetylated peptides were analyzed by MS are shown. **e** and **f** HuCCT1 cells were transfected with wildtype and mutant flag tagged cMYC plasmid and then treated with 10 μM RGFP966 for 24 h. The immunoprecipitated protein from cell lysates were analyzed via Western blotting and flag are shown as loading controls. **g** and **h** Flag-tagged cMYC and HA-tagged ubiquitin were coexpressed in HuCCT1 and HDAC3-knockdown cells, then treated with 10 μM RGFP966 for 24 h with or without 10 μM MG132. Ubiquitination levels of affinity purified Flag-cMYC proteins were detected and visualized by Western blotting. **i** HuCCT1 cells were transfected with HA tagged HDAC3 plasmid and then endogenous proteins were visualized by Western blotting

We then investigated whether the activity of HDAC3 could affect cMYC, since intracellular cMYC showed instability and is regulated by acetylation-related degradation at the K323 site in cancer cells. Moreover, HDAC3 inhibition decreases cMYC in a dose dependent fashion without altering its mRNA levels [11, 36]. We overexpressed cMYC in CCA cells, then purified the overexpressed protein by immunoprecipitation, and submitted them to both MS (mass spectrometry) as well as immunoblotting. We found that cMYC was physically intact with HDAC3 but not the other class I HDACs (HDAC 2 shares 52% identity with HDAC3 [26, 27]) (Fig. 6a). We inhibited cMYC synthesis by using CHX (Cycloheximide) and found that cMYC was degraded faster in the presence of a specific HDAC3 inhibitor (RGFP966) (Fig. 6b). Furthermore, when MG132 was included to inhibit proteasomal degradation, RGFP966-induced cMYC degradation was blocked (Fig. 6c). These results suggest that the ubiquitin-proteasome pathway mediates an acetylation-promoted decrease of cMYC [11]. To elucidate the direct target of HDAC3, we used an in vitro deacetylation system and found that both RGFP966 and pyruvate treatment inhibited HDAC3 deacetylation at the K323 site of cMYC (Fig. 6d). Consistent with MS results, HDAC3 inhibition enhanced acetylation of wildtype cMYC but resulting in marginal acetylation change after the lysine (K) of the K323 acetylation site was mutated to arginine (R) in CCA cells (Fig. 6e & f). Of note, the SIRT1 inhibitor EX527 could not change the acetylation of cMYC (Fig. 6d & Additional file 1: Figure S1C). Moreover, HDAC3 inhibition increased ubiquitination of cMYC, and conversely HDAC3 overexpression promoted endogenous cMYC levels in CCA cells (Fig. 6g, h & i). Together, these results suggest that HDAC3 inhibition enhanced the acetylation-promoted degradation of cMYC in CCA cells.

### HDAC3 inhibition induces CCA cell apoptosis by decreasing cMYC

**Fig. 7:**
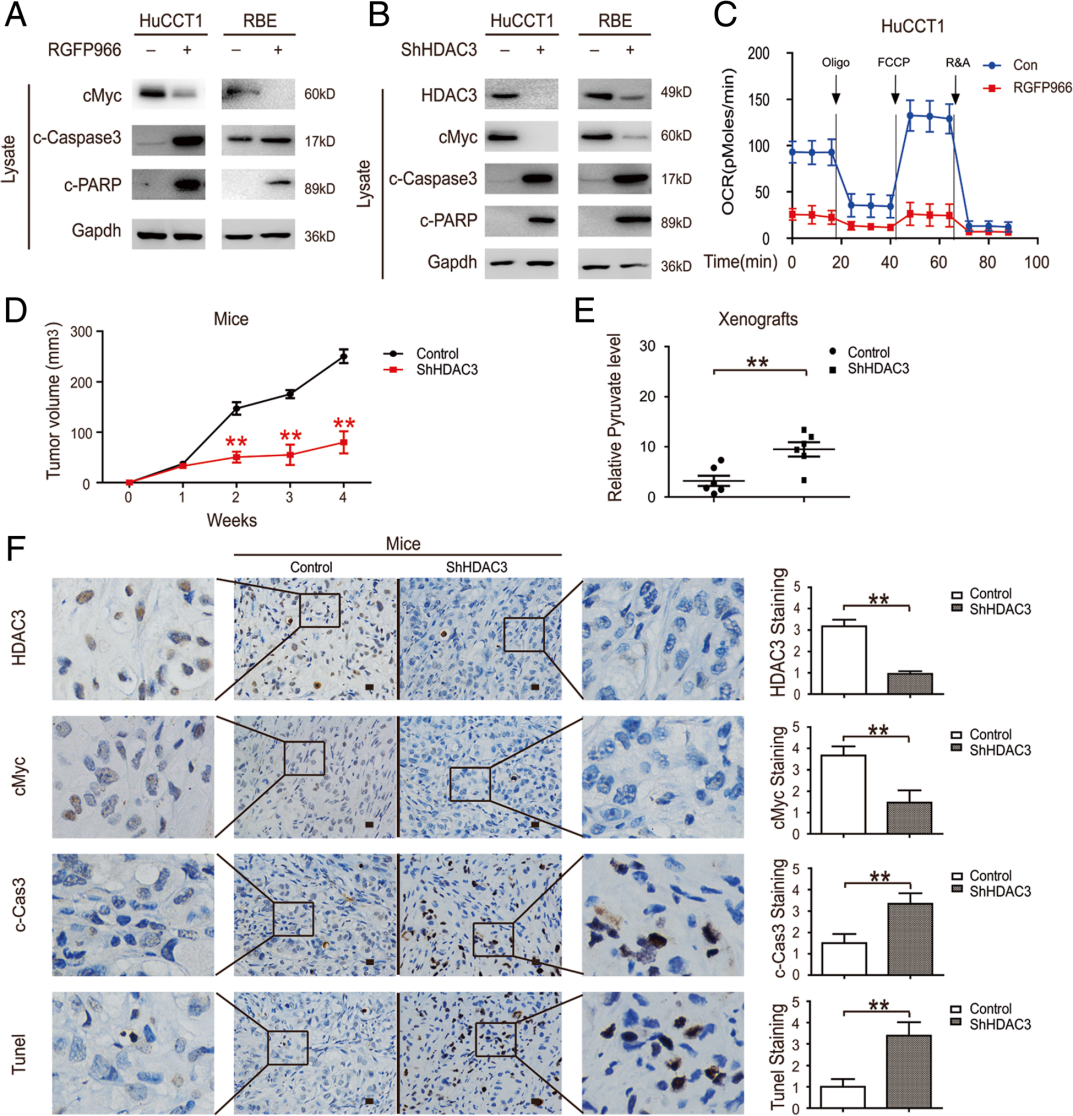
HDAC3 inhibition induces CCA cell apoptosis by decreasing cMYC. **a** HuCCT1 cells were treated with 10 μM RGFP966 for 24 h and then endogenous proteins were visualized by Western blotting. **b** HDAC3-knockdown HuCCT1 cells and their counterparts were lysated and then endogenous proteins were visualized by Western blotting. **c** The OCR of cells were detected at different time points after they treated with 10 μM RGFP966 for 24 h. **d** HDAC3 knockdown HuCCT1 cells and control counterparts were injected at the left and right sides of the same mice to induce xenograft tumor in nude mice. The xenograft tumor sizes were measured. **e** The pyruvate levels of xenograft tumor were measured. **f** The protein levels in the xenograft were detected (left) and quantified (right). Scale bars, 100 μm. Data represent the Mean ± SEM, n ≥ 3. *p < 0.05, **p < 0.01, NS not significant

Previous data confirmed that the accumulated pyruvate induced by cMYC inhibition promoted apoptosis in CCA cells, so we further inquired whether promoting cMYC degradation by HDAC3 inhibition could also induce CCA apoptosis. Both RGFP966 treatment and HDAC3 knockdown significantly decreased cMYC and increased caspase substrate (polyADP ribose polymerase (PARP) and caspase 3) cleavage; this effect was reversed by HDAC3 overexpression (Fig. 7a, b & Additional file 1: Figure S1D). Besides, oxygen consumption was extremely low after the treatment of RGFP966, which indicated that cells were on their way to apoptosis (Fig. 7c). Furthermore, HDAC3 knockdown significantly inhibited xenografts (Fig. 7d) and increased the pyruvate levels as well as decreased its biological targets (cMYC) in vivo (Fig. 7e & f). Importantly, HDAC3 knockdown induced high levels of pyruvate, which contributed to xenograft apoptosis (Fig. 7f). These results indicate that the apoptosis of CCA cells is primarily induced by HDAC3 inhibition, and cMYC-induced high levels of pyruvate may contribute to the process.

## Discussion

The Warburg effect represents a serious worldwide problem threatening the health of millions of cancer patients. However, how metabolites of the Warburg effect are beneficial for its induced-tumorigenesis, and what is the downstream target of these metabolites, still remains an unanswered question. Here, we set out to identify a promoting role for the low pyruvate levels as regulated by c-Myc and its dynamic acetylation in cancer cell proliferation. Low pyruvate levels contributed via downstream targets (PKM2 and LDHA) of c-Myc and attenuated the inhibition of HDAC3 as well as decreased HDAC3-regulated apoptosis. On the contrary, a high activity of HDAC3 stabilizes the cMyc protein by preferential deacetylation of cMyc at the K323 site, which further contributes to low pyruvate levels. This creates a positive feedback loop that promotes the Warburg effect and cell proliferation of the tumor.

It is well known that the dominant metabolite of the Warburg effect is lactate, and many previous studies focus on lactate and its role in tumor proliferation [37]. Since lactate is converted from pyruvate and the end product of enhanced glycolysis is pyruvate in cancer cells, we inquired whether this decreased pyruvate benefitted tumor proliferation. The underlying notion here is that decreased generation of pyruvate in CCA is the direct result of increased glycolysis and protects the tumor from apoptosis. We demonstrated an inhibitory effect of high levels of pyruvate on CCA cells, and it is noteworthy that intracellular pyruvate levels were negatively correlated with cell viability. This may be due to the fact that the expression of SLC5A8 (the gene coding for the Na + −coupled pyruvate transporter that regulates the entry of blood-borne pyruvate into cancer cells) is variant in CCA cells [28]. The glycolysis induced low pyruvate levels are due to both reduced production and excessive consumption. This reduced pyruvate production comes from the expression of pyruvate kinase, the enzyme responsible for the generation of pyruvate in glycolysis. We found the splice variant PKM2 (musclespecific pyruvate kinase 2) is expressed specifically in CCA with low catalytic activity. Moreover, we confirmed the high expression of LDHA in CCA, which is effective in the conversion of pyruvate into lactate and contributed to the predominant pyruvate consumption. Collectively, the high expression of PKM2 and LDHA maintained the low intracellular levels of pyruvate in cancer cells. Interestingly, inhibition of any one of these targets could neither induce tumor proliferation, nor reverse CCA metabolic type. Yet inhibition of these three targets increased pyruvate levels while promoting apoptosis.

Since pyruvate is an energy-rich nutrient necessary for growth in non-malignant cells, there must be an upstream regulator to maintain low levels of this metabolite in cancer cells. Our findings of the present study found that the upstream regulator of pyruvate is cMYC. As an oncogene, *c-Myc* has attracted extensive interest for its potential role in contributing to tumorigenesis. There are various mechanisms of cMYC-induced tumorigenesis, and an increased Warburg effect is one such mechanism. Many studies have also found that MYC increased metabolic proteins, such as LDHA and PKM2 [6, 7]. Thus, it has been considered a promising cancer target. Our studies demonstrate that cMYC is highly expressed in CCA and predicted a poor prognosis. Moreover, cMYC is positively correlated with PKM2 and LDHA, and contributed to the low pyruvate level. The stability of c-Myc protein is related to its acetylation at K323 [9, 10], and HDACi treatment, but not SIRTi treatment, induced c-Myc K323 acetylation as well as tumorigenesis inhibition [11, 12]. Employing immunoprecipitation, we further inquired as to which HDAC interacted with cMYC, and found that HDAC3 deacetylated cMYC at K323 and further protected cMYC from ubiquitinated degradation. Our work establishes cMYC inhibition as a strategy to accumulate pyruvate, which is effective in promoting apoptosis in CCA cells.

After confirming that the upstream regulator of pyruvate is cMYC, we moved to the other side and inquired as to what is the downstream target of pyruvate in CCA. Previous studies have concluded that the tumor suppressive function of pyruvate is related to its ability to inhibit HDACs; pyruvate is an HDAC inhibitor and a tumor suppressor [28]. Here we confirmed HDAC3 is the downstream target of pyruvate. It is important to note that HDAC3, which is capable of being inhibited by pyruvate, is up-regulated in CCA and protects CCA cells form apoptosis [18]. The elevation of HDAC3 activity is presumably necessary for the cancer cells to maintain their malignant phenotype [28]. Therefore, CCA cells must maintain low intracellular levels of pyruvate, lest HDAC3 will be inhibited and cell growth prevented by enhanced apoptosis.

**Fig. 8:**
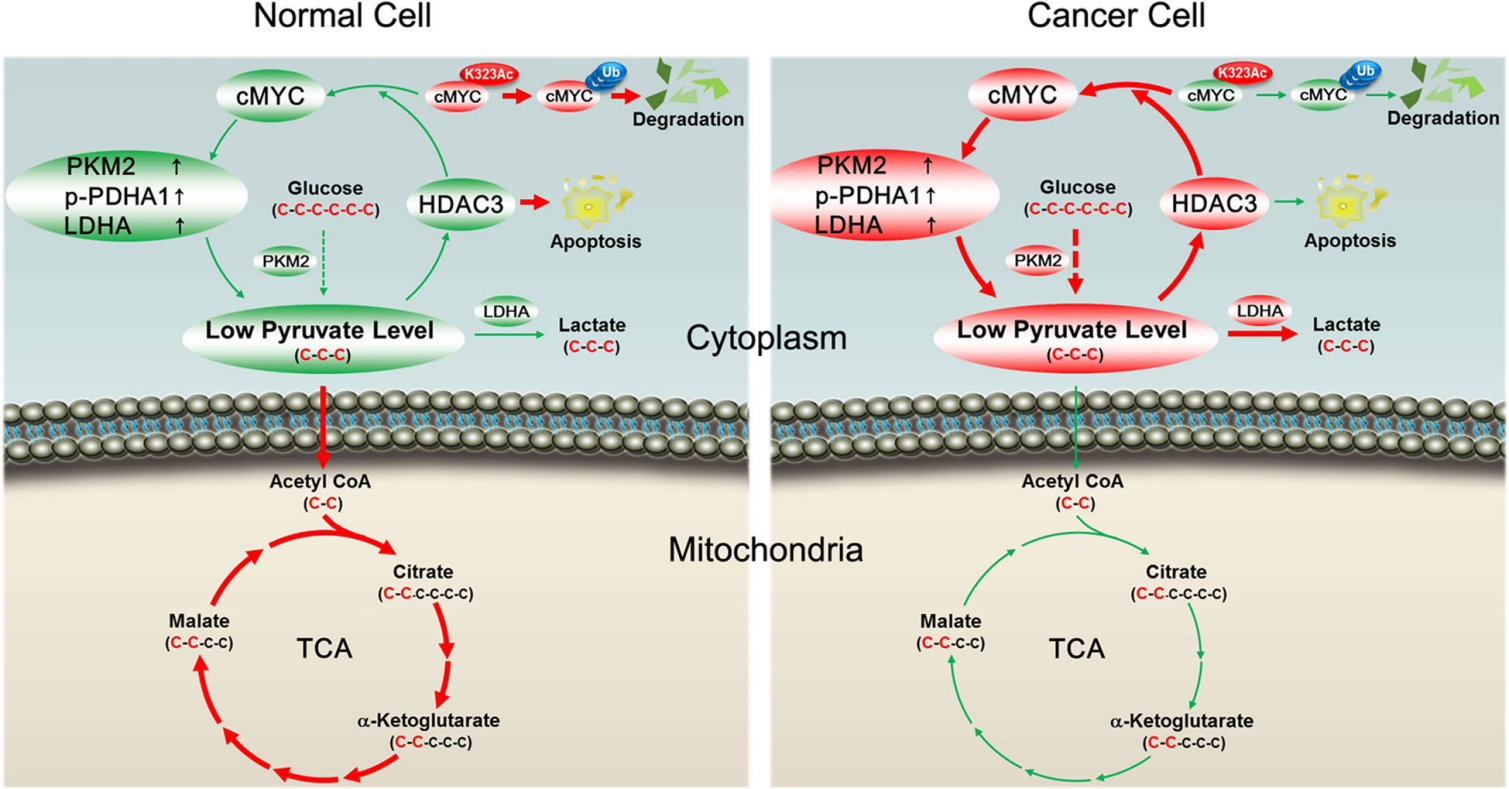
CCA cells consume glucose more avidly and convert it to lactate, resulting in a low pyruvate level, and this is important for cell proliferation. cMyc decreases pyruvate levels by promoting PKM2 and LDHA levels, consequently decreasing the inhibition to HDAC3 and protecting cancer cells from apoptosis. Conversely, high activity of HDAC3 stabilize the cMyc protein by preferential deacetylation of cMyc at the K323 site, which further contributes to low pyruvate levels. This creates a positive feedback loop that promotes the Warburg effect and cell proliferation of CCA

Deregulated apoptosis by histone acetylation is one of the mechanisms related to tumorigenesis [20]. Histone deacetylases (HDACs) catalyze the removal of acetyl groups from lysine tails. Since HDACs inhibit specific tumor suppressor genes, resulting in an aberrant epigenetic status and low apoptosis level of cancer cells, they may serve as candidate anti-cancer targets [38, 39]. Moreover, we have demonstrated that increased expression and activation of HDAC3 plays an anti-apoptotic role in epigenetic alterations, impacting pro-apoptotic pathways in CCA but not in normal cells [18]. Here, we demonstrated that cMYC-induced low intracellular levels of pyruvate decreased HDAC3 inhibition and promoted CCA proliferation. Interestingly, cMyc and HDAC3 constitutes a positive feedback loop, which is connected by pyruvate: cMyc decreases pyruvate levels by promoting PKM2 and LDHA levels, consequently decreasing inhibition to HDAC3 and protecting cancer cells from apoptosis. Conversely, high activity of HDAC3 stabilizes cMyc protein by preferential deacetylation of cMyc at the K323 site, which further contributes to the low pyruvate levels. This creates a positive feedback loop that promotes the Warburg effect and cell proliferation of the tumor (Fig. 8). To our knowledge, this is the first report describing this unique phenomenon of the positive feedback loop in cancer cells.

## Conclusions

In the present study, we set out to identify a promoting role for low pyruvate levels regulated by c-Myc and its dynamic acetylation in cancer cell proliferation. Moreover, we reported a unique phenomenon of the positive feedback loop with cMyc and HDAC3. Therapeutic strategies targeting pyruvate may be applicable to cancer treatment in a wide variety of tissues, since high expression of c-Myc is a common phenomenon in cancer.

## Additional file

Additional file 1: Low pyruvate levels protects cholangiocarcinoma. (A) The OCR of cells were detected at different time points after they treated with pyruvate and ethyl pyruvate for 48h. (B) cMYC protein levels in fresh tumor tissues from 25 CCA patients were analyzed by western blot. Then the correlation between the cMYC levels and their pyruvate levels were analysed and normalized to the protein level. C) HuCCT1 cells were transfected with flag tagged cMYC plasmid and then treated with 100 nM EX527 for 48 hours. The immunoprecipitated protein from cell lysates were analyzed via Western blotting and flag are shown as loading controls. (D) HDAC3-overexpressed cells and their counterparts were lysated, and then endogenous proteins were visualized by Western blotting. (TIF 1437 kb)

## Abbreviations

CCA: Cholangiocarcinoma
CHX: Cycloheximide
HDAC: Histone deacetylase
HDACi: HDAC inhibitiors
LDH: Lactate Dehydrogenase
MS: mass spectrometry
PARP: polyADP ribose polymerase
PKM2: Muscle specific Pyruvate Kinase 2
SIRT: sirtuins
