# Correction to: Low levels of pyruvate induced by a positive feedback loop protects cholangiocarcinoma cells from apoptosis

**DOI:** 10.1186/s12964-019-0357-z

**Published:** 2019-05-06

**Authors:** Mingming Zhang, Yida Pan, Dehua Tang, Robert Gregory Dorfman, Lei Xu, Qian Zhou, Lixing Zhou, Yuming Wang, Yang Li, Yuyao Yin, Bo Kong, Helmut Friess, Shimin Zhao, Jian-lin Wu, Lei Wang, Xiaoping Zou

**Affiliations:** 1 Department of Gastroenterology, Nanjing Drum Tower Hospital, the Affiliated Hospital of Nanjing University Medical School, Nanjing University, No.321 Zhongshan Road, 210008 Nanjing, People’s Republic of China; 2 Key laboratory of Reproduction Regulation of NPFPC (SIPPR, IRD), Fudan University, Shanghai, 200032 China; 3 Department of Digestive Diseases of Huashan Hospital, Shanghai, China; 4 School of Life Sciences, Fudan University, Shanghai, China; 5 Northwestern University Feinberg School of Medicine, Chicago, IL USA; 6 Department of Gastroenterology, Nanjing Medical University Affiliated Drum Tower Clinical Medical College, Nanjing Medical University, Nanjing, China; 7 Department of Surgery, Technical University of Munich (TUM), Munich, Germany; 8 State Key Laboratory of Quality Research in Chinese Medicine, Macau Institute for Applied Research in Medicine and Health, Faculty of Chinese Medicine, Macau University of Science and Technology, Avenida Wai Long, Taipa, Macao 442000 People’s Republic of China

**Correction to: Zhang et al. Cell Communication and Signaling (2019) 17:23**

**https://doi.org/10.1186/s12964-019-0332-8**

Following publication of the original article [1], the authors reported an error in Fig. 3. The corrected Fig. 3 is given below. Please note that these revisions do not affect the overall conclusions reported in the article. The authors apologise for the error and any inconvenience caused.

**Fig. 3:**
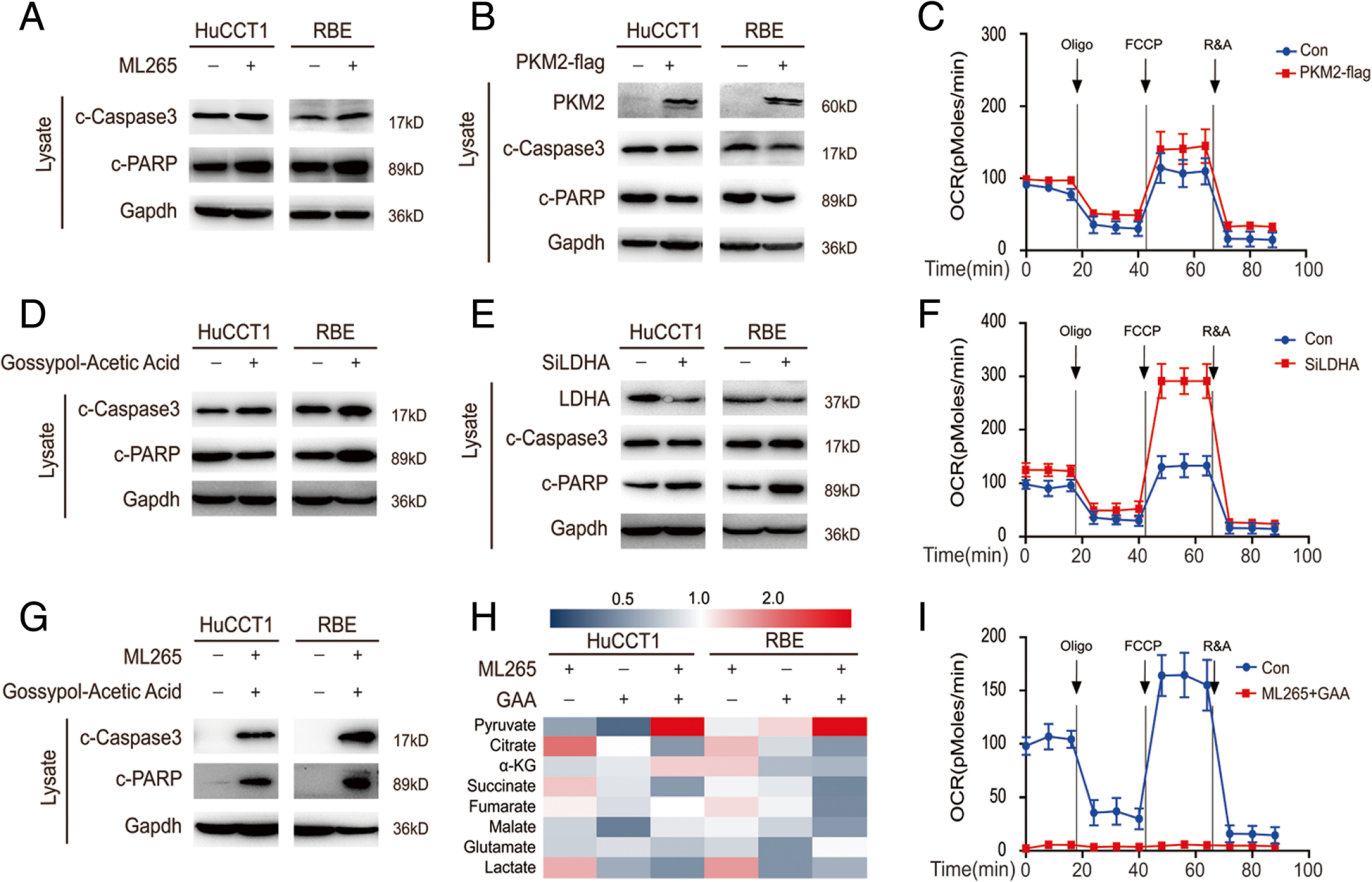
The changes in metabolic enzymes contribute to the low levels of pyruvate. **a** Cells were treated with 1 μM ML265 for 24 h and subjected to western blot. **b** PKM2 overexpressed cells and their counterparts were subjected to western blot. **c** The oxygen consumption rates (OCR) of PKM2-overexpressed cells and control cells were detected at different time points. OCR under oligomycin, carbonyl cyanide 4-(trifluoromethoxy) phenylhydrazone (FCCP), and antimycin A/rotenone treatments, respectively. **d** Cells were treated with 10 μM Gossypol-Acetic Acid (GAA) for 24 h and subjected to western blot. **e** LDHA knockdown cells and their counterparts were subjected to western blot. **f** The OCR of LDHA knockdown cells and control cells were detected at different time points. **g** Cells were treated with 1 μM ML265 and 10 μM GAA for 24 h, then subjected to western blot. **h** Cells were treated with 1 μM ML265 and 10 μM GAA for 24 h, the concentrations of the metabolites in GC cells were measured by Mass spectrometry (MS) and normalized to their protein level. **i** The OCR of cells were detected at different time points after they treated with 1 μM ML265 and 10 μM GAA for 24 h. Data represent the Mean ± SEM, *n* ≥ 3. *p < 0.05, **p < 0.01, NS not significant

## References

[1] Zhang (2019). Low levels of pyruvate induced by a positive feedback loop protects cholangiocarcinoma cells from apoptosis. Cell Commun Signal.

