# Supplementary figures and images for: Low levels of pyruvate induced by a positive feedback loop protects cholangiocarcinoma cells from apoptosis
Source: Cell Commun Signal. 2019 Mar 12;17:23. doi: 10.1186/s12964-019-0332-8 (PMC6417221; doi:10.1186/s12964-019-0332-8)

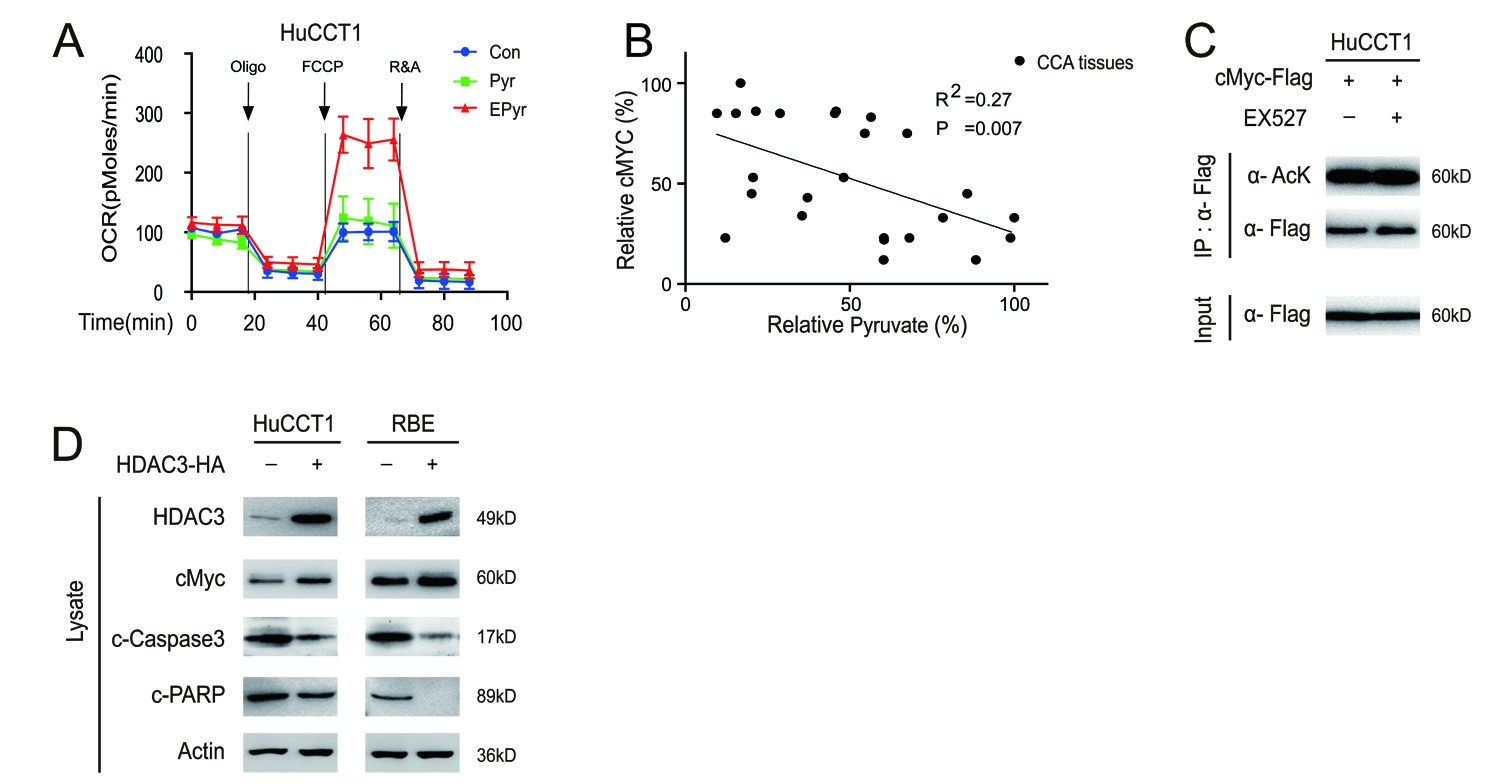

Supplement: Supplementary file 1 — Low pyruvate levels protects cholangiocarcinoma. (A) The OCR of cells were detected at different time points after they treated with pyruvate and ethyl pyruvate for 48h. (B) cMYC protein levels in fresh tumor tissues from 25 CCA patients were analyzed by western blot. Then the correlation between the cMYC levels and their pyruvate levels were analysed and normalized to the protein level. C) HuCCT1 cells were transfected with flag tagged cMYC plasmid and then treated with 100 nM EX527 for 48 hours. The immunoprecipitated protein from cell lysates were analyzed via Western blotting and flag are shown as loading controls. (D) HDAC3-overexpressed cells and their counterparts were lysated, and then endogenous proteins were visualized by Western blotting. (TIF 1437 kb) [file 12964_2019_332_MOESM1_ESM.tif]
